# Evaluation of Quality and Storage Characteristics of Freeze-Dried Powdered Mycelium Sausages According to Packaging Methods

**DOI:** 10.3390/foods14234080

**Published:** 2025-11-27

**Authors:** Yu-Na Oh, Hack-Youn Kim

**Affiliations:** 1 Department of Animal Resources Science, Kongju National University, Yesan 32439, Republic of Korea; qkffpfls7624@naver.com; 2 Resources Science Research Institute, Yesan 32439, Republic of Korea

**Keywords:** *Lentinula edodes*, shiitake mushroom, mycelium, soybean, shiitake mushroom mycelium sausage, packaging, storage

## Abstract

With the increases in the global population, demand for meat, and income, plant-based meat alternatives using mushrooms, soybeans, and other ingredients are attracting increasing attention to address food security. Mushroom mycelia have a high moisture content and are prone to quality deterioration; therefore, interventions, such as freeze-drying and optimized packaging, are necessary to improve shelf life. Furthermore, controlling storage temperature is a key factor in preserving product quality. Therefore, in this study, *Lentinula edodes* (shiitake mushroom) mycelium sausage were stored under various packaging methods (aerobic packaging, vacuum packaging, and modified atmosphere packaging) and storage temperatures (0 and 4 °C). Quality and storability were evaluated at 0, 7, 15, 30, and 50 days using tests for proximate components, pH, storage loss, color, water activity (Aw), aerobic bacterial count, thiobarbituric acid reactive substances (TBARS), and volatile basic nitrogen (VBN) using an electronic nose and an electronic tongue. The vacuum-treated groups showed superior pH, CIE L*, CIE a*, Aw, TBARS, and VBN levels. At 0 °C, each treatment group exhibited significantly lower VBN levels on days 30 and 50 (*p* < 0.05). Overall, vacuum packaging and low storage temperatures are expected to be beneficial for maintaining the quality and storage characteristics of shiitake mushroom mycelium sausages.

## 1. Introduction

With the increase in life expectancy and income, the global population is projected to reach 10 billion by 2050 and meat demand is expected to increase by 70%. This has made food security a pressing global challenge [1]. To meet this growing demand, various alternative meat products, including plant-based meat substitutes prepared from soybeans, mushrooms, seaweeds, insect proteins, and cultured meat, are gaining attention [2]. Over the past 15 years, advancements in food manufacturing technologies have improved the sensory qualities of meat alternatives, and increasing awareness of environmental and ethical issues is expected to further drive the growth of the alternative meat market [3]. Among these, plant-based meat substitutes demonstrate high consumer acceptance and are considered to have significant growth potential [4]. Fungus-based meat alternatives can effectively mimic the appearance, flavor, and texture of conventional meat, and their low allergenic potential makes them a promising protein source for the future [5].

Mushroom mycelia enable rapid cultivation and eco-friendly production and can serve as a nutritional alternative to animal protein. However, their high moisture content makes it prone to spoilage, necessitating measures to extend its shelf life [6]. Methods for extending the shelf life of mushrooms include hot-air-drying and freeze-drying. Freeze-drying allows powdering while maintaining mushroom quality, facilitating easy addition during meat product manufacturing [7]. In addition, freeze-drying helps preserve the nutritional properties of the product while inhibiting microbial spoilage, thereby maintaining overall product quality [8]. Another approach to extending the shelf life involves packaging methods, which contribute to maintaining product freshness by inhibiting microbial growth and biochemical reactions [9]. These effects can be achieved by controlling the packaging methods (such as aerobic packaging, vacuum packaging, and modified atmosphere packaging) and storage temperatures [9].

Stretched polystyrene containers are primarily used in aerobic packaging. Because air permeates these materials, they have limitations in extending their shelf life, with sensory characteristics deteriorating within 3–4 days [10]. Vacuum packaging is a method of extending the shelf life of food by removing O_2_ from the packaging material, creating an environment that makes it difficult for aerobic microorganisms to grow, and thus improving microbial stability [10,11]. Modified atmosphere packaging (MAP) is a storage technique in which a controlled mixture of gases is introduced into the package, typically consisting of O_2_, N_2_, and CO_2_. Oxygen plays an important role in maintaining the desirable red color of meat; however, higher O_2_ levels can accelerate oxidative reactions, and thus inert N_2_ is commonly used to regulate the oxygen concentration within the package [12]. Meanwhile, CO_2_ plays an important role in extending shelf life by inhibiting oxidative reactions as well as suppressing the growth of aerobic bacteria and molds [13].

Although numerous studies have examined the production of meat analogs using fungal mycelium and evaluated their storage properties, most of these investigations have focused on comparing formulations with different mycelium ratios [14,15,16]. Therefore, the present study employed a fixed formulation consisting of freeze-dried shiitake mushroom mycelium powder and plant proteins to produce sausage-type meat analogs, and subsequently evaluated their storage behavior under different packaging methods and storage temperatures. Subsequently the quality characteristics and storage stability of the shiitake mushroom mycelium sausages (SMMS) over time, this study aims to identify optimal packaging conditions and storage temperatures that can extend the product’s shelf life.

## 2. Materials and Methods

### 2.1. Shiitake Mushroom Mycelium Culture

The shiitake mushroom mycelia were provided by Ohsooup Co., Ltd. (Gangneung, Republic of Korea) and were cultured using the method described by Choi et al. [17]. No genetic or morphological identification was performed on the provided shiitake mushroom mycelium. Sterile fruiting body tissue was inoculated onto PDA (potato dextrose agar) solid medium and cultured at 25 °C for 20 days to obtain the mycelium. For the shiitake mushroom mycelium inoculum culture, a portion of the shiitake mushroom mycelium cultured on PDA was inoculated into 300 mL of PDB (potato dextrose broth) liquid medium. This was homogenized using a high-speed homogenizer at 10,000 rpm for 40–60 s, then cultured for 10 days in a shaking incubator (temperature 25 °C, at 25 °C, shaking speed 250 rpm, air supply 0.5 vvm). For mass production, liquid medium was prepared by adding yeast extract 0.5% (*w*/*v*), glucose 2.0% (*w*/*v*), MgSO_4_·7H_2_O 0.05%, KH_2_PO_4_·12H_2_O 0.05% (*w*/*v*), and silicon-based antifoaming agent (LS-303) 0.05% was prepared. Mycelium was recovered by vacuum filtration using a filter paper with a pore size of 10 μm or less and washed three times with distilled water to remove residual medium components.

### 2.2. Freeze-Drying Shiitake Mushroom Mycelium and Sausage Production

The freeze-drying conditions of shiitake mushroom mycelium were based on the method described by Choi et al. [18], and the freeze-drying time was increased until the shiitake mushroom mycelia were powdered without clumping. The shiitake mushroom mycelium was freeze-dried using a freeze dryer (FD12008, ilShinBioBase, Yangju, Republic of Korea) at a temperature of −121 °C and a vacuum pressure of 5 mTorr for 170 h. It was then pulverized using a vacuum mixer (HR3752/00, Philips, Amsterdam, The Netherlands) for 1 min each. The ground powder was first sieved using a standard sieve No. 18 (DH.Si8031, DAIHAN Scientific, Wonju, Republic of Korea), then finally sieved through a standard sieve No. 35 (DAIHAN Scientific, Wonju, Republic of Korea) to standardize it to 32 mesh for use in sausage production. A sausage dough gel was prepared by blending 20% shiitake mushroom mycelium powder, 30% isolated soy protein, 40% wheat gluten, 10% corn starch, and 1.5% salt. No emulsifiers or additional binders were added to the formulation. The final gelatinized mixture was prepared by mixing twice for 1 min each using a Bowl cutter (K-30, Talsa, Valencia, Spain). The prepared gelatinized mixture was then filled into cellulose casings using a stuffer (EM-12, Mainca, Barcelona, Spain). Subsequently, the mixture was heated in a chamber (10.10ESI/S, Alto Shaam Co., Menomonee Falls, WI, USA) at 80 °C until the core temperature reached 75 °C, followed by cooling at room temperature for 30 min. The samples were then packaged using three different methods: Control (shrink packaging with 78% N_2_ and 20% O_2_), vacuum packaging, and MAP (60% CO_2_ and 40% O_2_). The gas composition for the MAP treatment was determined based on the maximum antimicrobial effectiveness of CO_2_ reported by Liang et al. [19], with the remaining 40% O_2_ included to allow discrimination among treatments and to monitor oxidative changes. Storage temperatures were 0 °C and 4 °C, with samples used at 0, 7, 15, 30, and 50-day intervals. For the Day 0 samples, the pre-treatment results were applied identically to all treatment groups.

### 2.3. Proximate Composition

The general composition of SMMS was measured according to AOAC methods (2016). Moisture content was determined by atmospheric drying (AOAC 950.46), crude protein content by the Kjeldahl method (AOAC 992.15), crude fat content by the Soxhlet method (AOAC 960.39), and crude ash content by the direct combustion method (AOAC 920.153), and expressed as percentages.

### 2.4. pH

SMMS and distilled water were mixed at a 1:4 ratio and homogenized for 1 min using an Ultra-Turrax (HMZ-20DN, Pooglim Tech, Gunsan, Republic of Korea) set at 6451× *g*. The pH was then measured using a pH meter (Model S220, Mettler-Toledo, Columbus, OH, USA) calibrated with pH 4.01, pH 7.00, and pH 10.00 buffer solutions (Suntex Instruments, New Taipei City, Taiwan).

### 2.5. Storage Loss

After manufacturing SMMSs, samples were weighed to a uniform 5 g. Control, vacuum-packed, and MAP-packed samples were stored at 0 °C and 4 °C, while retort-packed samples were stored at room temperature. Weight changes were measured at 1, 7, 15, 30, and 50 days. Storage loss was calculated using the following equation, applying the measured weight at day 0 and the weight changes over the storage period:

$$Storage\;loss\;(\%)=\frac{A-B\;(g)}{A\;(g)}\times 100$$

A = Weight on Day 0
B = Weight by storage period (7, 15, 30, 50 days).

### 2.6. Color

The brightness (CIE L*), redness (CIE a*), and yellowness (CIE b*) of the cross-section of SMMS were measured using a colorimeter (CR-10, Minolta). The standard color was a white standard plate with CIE L* value set to +97.83, CIE a* value to −0.43, and CIE b* value to +1.98.

### 2.7. Water Activity (Aw)

SMMS was ground using a hand blender (HR-2652; Philips, Amsterdam, The Netherlands) to produce a uniform mixture. Subsequently, approximately 14 g of the ground sample was placed in a dedicated container, weighed, and its water activity measured using a water activity meter (LabMaster-aw NEO, Novasina AG, Zurich, Switzerland).

### 2.8. Aerobic Plate Count (APC)

The APC of SMMS was measured according to the following method. To 10 g of sample, 20 mL of 0.1% buffered peptone water (BPW) was added, and the mixture was homogenized for 1 min using a stomacher (WH4000-2751-9, 3M, Saint Paul, MN, USA). Then, 1 mL of the filtrate was diluted in 9 mL of 0.1% BPW, and this process was repeated as many times as necessary. The diluted filtrate was spread onto tryptic soy agar plates and incubated at 37 °C for 24 h in an incubator (WSC-2610, ATTO, Tokyo, Japan). The number of colonies formed was counted and expressed as log colony forming units (log CFU/g).

### 2.9. Thiobarbituric Acid Reactive Substances (TBARS)

TBARS in SMMSs were measured using an extraction method. After adding 12.5 mL of distilled water, 12.5 mL of 10% perchloric acid (PCA), and 200 μL of 0.3% butylated hydroxytoluene (BHT) were added to 5 g of sample. The mixture was homogenized for 1 min at 5614× *g* using a homogenizer (AM-5, Nihonseiki Kaisha Ltd., Akita, Japan). The mixture was then filtered through filter paper to extract the supernatant. The supernatant was mixed with 0.02 M 2-thiobarbituric acid in a 1:1 ratio and reacted for 10 min in a water bath set at 100 °C. Absorbance was then measured at 532 nm using a multi-mode microplate reader. A standard curve was prepared using 1,1,3,3-tetraethoxypropane, and the results were expressed as mg MDA/kg (MDA: malondialdehyde).

### 2.10. Volatile Basic Nitrogen (VBN)

Add 30 mL of distilled water to 10 g of SMMS, then homogenize using a homogenizer at 5614× *g* for 1 min. Transfer the homogenate to a measuring cylinder, add distilled water to the 100 mL mark, and filter through filter paper. Add 1 mL of the filtrate to the outer chamber of the Conway vessel. Place 1 mL of 0.01 N H_3_BO_3_ and 100 µL of Conway reagent into the inner chamber. To prevent vaporization, slightly open the lid and add 1 mL of 50% K_2_CO_3_to the outer chamber. The main body and manual lid were secured with a clip to ensure a tight seal, and the mixture was reacted at 37 °C for 2 h. After reacting at 37 °C for 2 h, the amount of nitrogen volatilized and captured in H_3_BO_3_ was measured by titration with 0.02 N H_2_SO_4_. This value was then substituted into the calculation formula below to determine the VBN.

$$VBN\;(mg\%)=\frac{V1-V2}{m}\times 0.14\times a\times b\times 100$$

V1: Sample titration volume (mL);
V2: Titrant volume (mL);
m: Sample mass (g);
*a*: 0.02 N H_2_SO_4_ titration strength;
*b*: Dilution factor.

### 2.11. Electronic Nose (E-Nose)

A vial containing 2 g of SMMS was measured using an electronic nose (HERACLES-2-E-NOSE, Alpha MOS, Toulouse, France) equipped with two columns, MXT-5 (Restek, Bellefonte, PA, USA) and MXT-1701 (Restek), under 70 °C conditions. Data were analyzed using the Alphasoft program (Alphasoft, Alpha MOS) under the following conditions: response time 110 s, injection volume 5000 μL, injection rate 125 μL/s, injection port temperature 200 °C, detector temperature 260 °C.

### 2.12. Electronic Tongue (E-Tongue)

SMMS and distilled water were mixed at a 1:5 ratio and homogenized for 1 min using an Ultra-Turrax (HMZ-20DN, Pooglim Tech) set at 6451× *g*. The mixture was then centrifuged at 437× *g* for 10 min at 4 °C using a centrifuge (Supra R22, Hanil, Daejeon, Republic of Korea) to extract the supernatant. The supernatant, diluted 1000-fold with distilled water, was measured using an electronic tongue system (Astree 5, Alpha MOS, Toulouse, France). The CTS (for saltiness), NMS (for umami), and AHS (for sourness) sensors, along with PKS and ANS (standard sensors) and SCS and CPS (auxiliary sensors), to confirm the signal intensity of the taste components indicated by each sensor. CTS, NMS, AHS, PKS, ANS, SCS and CPS refer to specific sensor types within the e-tongue system, rather than abbreviations.

### 2.13. Statistical Analysis

All experiments were repeated at least three times, and results were analyzed. SAS (version 9.4 for Windows, SAS Institute Inc.; statistical processing program) was used to present the mean values and standard deviations of the results. Two-way analysis of variance was performed on the experimental results, and Duncan’s multiple range test was used to verify significant differences (*p* < 0.05).

## 3. Results and Discussion

### 3.1. Proximate Composition

The results of the general components of SMMSs according to packaging method, storage temperature, and duration are shown in Table 1. Moisture content showed a decreasing trend as the storage period progressed, with all treatment groups exhibiting a significant decrease at 50 days compared to that at 0 days (*p* < 0.05). The protein content showed no significant differences among the pretreatment groups based on the temperature. It exhibited an increasing trend over the storage period, significantly increasing on day 50 compared to that on day 0 (*p* < 0.05). Ash content showed no significant differences among the pretreatment groups based on the packaging method, storage temperature, or duration (*p* > 0.05). Fat content was not detected in the pre-treatment group or as the storage duration increased. As the pH of the product approaches its isoelectric point, the electrostatic repulsion between protein molecules decreases, reducing protein solubility and weakening the gel structure, which in turn reduces the water-holding capacity [20,21]. The isoelectric point of soybean protein is pH 4.5, while the isoelectric points of wheat proteins gliadin and glutenin are reported as pH 5.5 and pH 4.6, respectively [22,23]. In this study, the pH of the pretreated samples decreased to <6 as the storage period progressed. Consequently, as the pH approached the isoelectric point of soybean protein and wheat gluten, the water-holding capacity decreased, leading to reduced moisture content. These results are similar to those of Park et al. [24] who found that a lower water-holding capacity is correlated with a lower moisture content. Meanwhile, protein content can increase as moisture content decreases [25]. Therefore, in this study, we determined that protein content increased as moisture content decreased over the storage period. Oh and Kim [26] reported a negative correlation between moisture and protein content in mushroom mycelium sausages, similar to the findings of this study. Macro- and microelements are less affected by storage duration and temperature; therefore, their content remains relatively stable regardless of storage conditions [27]. Accordingly, the ash content of SMMSs did not show significant differences based on various packaging methods, storage temperatures, or storage periods. Naveed et al. [28] reported that the ash content of brown rice did not show significant differences under various storage temperatures and periods, which is similar to this study. Meanwhile, during Soxhlet extraction, incomplete lipid recovery may occur when lipids are bound to proteins, resulting in inaccurate measurements. In addition, because the method quantifies lipid content based on the weight of the extracted residue, samples with very low fat levels may yield values below the detectable range [29]. Therefore, the absence of detectable lipids in this study is likely attributable to both the methodological limitations of the Soxhlet procedure and the inherently low fat content of the raw shiitake mushroom mycelium (0.32%) used in the formulation [30].

### 3.2. pH and Storage Loss

The results of pH and storage loss for SMMSs according to packaging method, storage temperature, and duration are shown in Table 2. At 30 days of storage, significantly lower pH values were observed in the MAP 0 °C and 4 °C treatment groups (*p* < 0.05). Furthermore, the pH tended to decrease over the storage period in all pretreated groups, showing a significantly lower value on day 50 than on day 0 (*p* < 0.05). The lower pH values in the MAP-treated groups were associated with increased CO_2_ concentration. During storage, SMMSs absorb CO_2_, producing H_2_CO_3_, which can decrease the pH [31]. Faisal et al. [32] reported a decrease in pH when meat was MAP-packed, attributed to CO_2_ dissolving in the product’s moisture and the formation of H_2_CO_3_, showing similarity to this study. Furthermore, the reaction of CO_2_ with internal moisture in proteins to form H_2_CO_3_, ultimately leading to H^+^ generation and a decrease in pH, can similarly occur in plant proteins [33]. Mustafa and Andreescu [34] reported that CO_2_ reacts with moisture in grains to produce H_2_CO_3_, supporting the possibility that the decrease in pH observed in this study was due to CO_2_ within the MAP. Meanwhile, as the storage period elapses, microorganisms use proteins and sugars for metabolic activities, producing organic acids such as lactic acid and acetic acid, which may lower the pH of the product [35,36]. In this study, the total bacterial count also showed an increasing trend as the storage period progressed. Consequently, it was determined that the pH decreased because of the production of organic acids from microbial metabolic activity. Uysal et al. [37] reported that the pH decreased owing to lactic acid bacterial activity as the storage period progressed, showing results similar to those of this study.

In terms of storage loss, the control treatment showed a lower tendency for storage loss than other treatments, while the MAP 4 °C treatment exhibited a significantly higher storage loss (*p* < 0.05). Within the same packaging method, changes due to temperature showed a higher tendency at 4 °C than at 0 °C. Furthermore, all treatments exhibited a tendency for storage loss to increase over the storage period. Vacuum packaging can cause drip loss owing to moisture exudation from the product under applied pressure [38]. Similarly, during MAP, increased CO_2_ concentration and accumulation can lead to higher drip loss [39]. In addition, the presence of CO_2_ and oxidative conditions can disrupt protein structures, and H_2_CO_3_ formed from dissolved CO_2_ can induce protein denaturation, thereby reducing water-holding capacity and increasing drip loss [40]. Reyes et al. [41] reported increased drip loss in beef during vacuum packaging due to moisture exudation during sealing, whereas Holck et al. [42] found that higher CO_2_ concentrations in MAP-packaged chickens increased drip loss, which is consistent with the results of the present study. Drip loss is an economically relevant parameter that is negatively correlated with water-holding capacity and positively correlated with storage duration [43]. As the storage temperature increases, the water-holding capacity of food decreases; conversely, lower temperatures during refrigerated storage maintain product quality characteristics more stably [22]. Furthermore, weakening of gel bonds during storage leads to structural deterioration, reduced water-holding capacity, and increased drip loss [44]. Augustyńska-Prejsnar et al. [25] reported higher storage loss over time at 6 °C than at 2 °C when storing chicken meat, whereas Miller et al. [45] observed increased storage loss with an extended storage duration in plant-based patties, which is consistent with the findings of the present study. Accordingly, it was determined that water loss increased as the water-holding capacity decreased with storage temperature at 4 °C and the passage of storage time. Notably, the MAP treatment at 4 °C showed a significantly higher storage loss, likely resulting from protein structural disruption and denaturation induced by the presence of CO_2_.

### 3.3. Color

The color results of shiitake mushroom mycelium-containing sausages according to the packaging method, storage temperature, and duration are shown in Table 3. For CIE L*, significantly higher values were observed at 30 and 50 days in the order of vacuum, MAP, and control (*p* < 0.05). Furthermore, the lightness of the pretreated samples decreased significantly from day 0 to 7 and from day 50 (*p* < 0.05). For CIE a*, significantly higher values were observed at 50 days in the control 4 °C and MAP 4 °C treatments, while significantly lower values were found in the vacuum-treated group at both 30 and 50 days (*p* < 0.05). Furthermore, as the storage period progressed, the control and MAP-treated groups showed a tendency toward increased redness, whereas the vacuum-treated group showed no significant difference (*p* > 0.05). For CIE b*, the MAP-treated group showed significantly higher values on days 7, 15, 30, and 50 (*p* < 0.05) and did not show significant differences as the storage period progressed (*p* > 0.05). For the control and vacuum-treated groups, CIE b* decreased significantly on day 7 compared to that at day 0, but no significant differences were observed during the remaining storage periods (*p* > 0.05). The Maillard reaction is a reaction between reducing sugars and amino acids or peptides that occurs more actively at high protein concentrations [46]. When melanoidins are formed through the Maillard reaction, browning progresses, potentially darkening the color of food. If the Maillard reaction occurs during storage, the lightness may decrease [47,48]. Meanwhile, shiitake mushroom mycelia are rich in L-phenylalanine, which is involved in the biosynthesis of phenolic compounds, as well as various phenolics such as caffeic acid and vanillic acid [49]. In foods, browning is primarily associated with enzymatic reactions between phenolic compounds and polyphenol oxidases present in fruits and vegetables. Physical and mechanical stimuli, temperature changes, and oxidative processes lead to the formation of quinones from phenolics, which are subsequently converted into melanins, resulting in a brown coloration [50]. Cook et al. [22] found decreased lightness and no significant difference in yellowness with storage time, consistent with the results of the present study. Accordingly, this study also concluded that lightness decreased and redness increased through the Maillard reactions and phenol browning that occurred during the storage period. Vacuum packaging plant-based meat alternatives effectively suppresses color changes during storage by inhibiting oxidation under low-oxygen conditions [51,52]. Furthermore, O_2_ presence during packaging promotes drip loss and lipid oxidation in the product, thereby reducing the color stability [53]. Therefore, the control and MAP treatments, which contained O_2_, exhibited lower color stability than the vacuum-treated group.

### 3.4. Aw and APC

The water activity and APC results for shiitake mushroom mycelium-containing sausages according to the packaging method, storage temperature, and duration are presented in Table 4. For water activity, within the same packaging method, water activity at 4 °C tended to be higher than that at 0 °C. As the storage progressed, the water activity of the pretreated samples increased. Water activity refers to the amount of water available to microorganisms for metabolism [54]. Because water activity is an intrinsic factor in food spoilage by microorganisms, it is important to lower water activity to ensure food stability [55]. Foods with higher water activity become more susceptible to spoilage as microbial growth becomes more active, and enzymatic reactions are stimulated [56]. Furthermore, according to the Arrhenius equation, where reaction rates increase with increasing temperature, the water activity is lower at lower temperatures [57]. Therefore, the temperature of 0 °C, which exhibits significantly lower water activity, is considered suitable for maintaining the quality of plant-based sausages during storage. Protein oxidation and degradation during storage as the protein structure denatures, bound and fixed water decrease, while free water increases [58]. Therefore, as the storage period progressed, the free water increased, leading to an increase in water activity. Corrêa et al. [59] reported that the water activity of plant-based sausages was approximately 0.97, making them susceptible to microbiological spoilage. However, in this study, the pretreated group exhibited a water activity of approximately 0.84, even on day 50, suggesting safety from foodborne pathogen proliferation and quality deterioration.

For APC, no significant differences were observed between treatment groups until days 7 and 15 (*p* > 0.05), but significantly higher APC levels were observed in the control 4 °C group at days 30 and 50 (*p* < 0.05). The MAP-treated group showed significantly lower APC at 30 days (*p* < 0.05), and at 50 days, no significant differences were observed between the treatment groups, except for the control 4 °C group (*p* > 0.05). Furthermore, the APC in all treatment groups increased significantly at 50 days compared to that at 0 days (*p* < 0.05). Food packaging methods and storage temperatures influence microbial proliferation during storage, leading to food spoilage via microbial growth and metabolic activity [60]. Vacuum packaging suppresses the availability of oxygen and moisture necessary for microbial growth by sealing the food, whereas MAP enhances the microbiological stability of food through the bacteriostatic effect of CO_2_ within the packaging [19,61]. In contrast, aerobic packaging has a shorter shelf life than vacuum and MAP because it is less effective in maintaining food quality [62]. Neo et al. [53] reported that aerobic packaging is more susceptible to microbial growth than vacuum packaging, and Yim et al. [63] reported that MAP is superior to vacuum packaging in inhibiting microbial growth; these findings are similar to those of this study. Meanwhile, microbial growth and metabolic activity are more favorable at lower storage temperatures, as lower temperatures delay food spoilage, thereby improving shelf life [64]. Cook et al. [22] measured APCs in plant-based meat alternatives stored at 1 °C, 4 °C, and 7 °C for 15 days. They reported the highest APC at 7 °C during the 15-day storage period, which is consistent with the findings of this study. Accordingly, a high APC was observed in the control group at 4 °C during the 50-day storage period. Thus, vacuum packaging and MAP exhibited low APCs due to their microbial suppression effects. Plant-based meat alternatives typically exhibit high water activity and slightly acidic pH, making them susceptible to microbial spoilage depending on storage conditions [65,66]. Kabisch et al. [66] conducted experiments on ten types of plant-based meat alternatives and reported that the mean total aerobic plate count (APC) of mesophilic bacteria was 4.33 log_10_ CFU/g, with lactic acid bacteria accounting for the majority at 3.75 log_10_ CFU/g. Additionally, Geeraerts et al. [67] measured total bacterial counts in six types of plant-based meat products past their shelf life and found a wide range from below 2 log CFU/g to 8.7 log CFU/g, indicating progression toward spoilage. These findings suggest that microbial growth and spoilage patterns vary depending on the type of plant-based meat, highlighting the need for further studies on the microbiological characteristics of SMMSs.

### 3.5. TBARS and VBN

The TBARS and VBN results for shiitake mushroom mycelium-containing sausages according to the packaging method, storage temperature, and duration are presented in Table 4. For TBARS, the values tended to be the highest in the order of MAP, control, and vacuum on day 30. On days 7, 15, 30, and 50, the MAP-treated group showed significantly higher values (*p* < 0.05). Furthermore, the TBARS content tended to increase in all pretreated groups as the storage period progressed. TBARS assessed the degree of food rancidity by analyzing the pink color produced through the TBA-MDA reaction at an absorbance of 532 nm, using MDA, a secondary decomposition product resulting from lipid oxidation, as an indicator [68]. Oxidative rancidity degrades quality attributes, such as nutritional value, flavor, and color, reduces storage stability, and shortens shelf life. Therefore, inhibition of lipid oxidation significantly affects consumer acceptance [69]. Reactive oxygen species (ROS) attack the cell membranes of mycelia, leading to the oxidation of lipid components and an increase in TBARS content [70]. Soy protein isolates also contain lipids [71], which are primarily rich in unsaturated fatty acids such as oleic acid, linoleic acid, and α-linolenic acid [72]. Lipid peroxidation is closely related to the presence of unsaturated fatty acids, and lower lipid content generally corresponds to lower TBARS values [73]. Accordingly, lipid oxidation occurred in both shiitake mushroom mycelium and ISP, and the low TBARS levels observed in SMMSs are likely due to their low lipid content. Reducing the O_2_ concentration within the packaging helps inhibit food oxidation [74]. Li et al. [75] measured TBARS in pork based on packaging methods and reported that after 14 days, the MAP treatment containing 40% O_2_ showed higher TBARS content than vacuum packaging, which is similar to the results of this study. Additionally, Lee and Kang [76] reported that higher O_2_ concentrations during MAP promoted lipid oxidation, leading to an increased TBARS content. Accordingly, the MAP (O_2_ 40%) treatment group showed higher TBARS content than the control (O_2_ 20%) group, while the oxygen-suppressed vacuum-packed group exhibited lower TBARS content.

For VBN, no significant differences were observed between the pretreatment groups on days 7 and 15 (*p* > 0.05). At days 30 and 50, VBN content was significantly higher in the MAP, control, and vacuum-treated groups, respectively, in that order, depending on the storage temperature (0 °C or 4 °C), showing the same trend under both temperature conditions (*p* < 0.05). Furthermore, the VBN content tended to increase over the storage duration in all the pretreated groups. VBN is the accumulation of volatile amines produced when proteins or nitrogen-containing compounds are degraded by endogenous enzymes and microbes in food. As storage progresses, increasing VBN levels degrade the food’s sensory characteristics [77,78]. Furthermore, at low storage temperatures, microbial metabolic activity is inhibited, delaying VBN production, while increased O_2_ concentration within the package promotes protein oxidation and deterioration in the food, thereby accelerating VBN formation [79,80]. Accordingly, MAP (O_2_ 40%) showed higher VBN content than the control (O_2_ 20%) treatment, and VBN content was higher at 4 °C than that at 0 °C. Liang et al. [19] reported that storing lambs at various temperatures promotes VBN production at higher storage temperatures. Similarly, Liang et al. [80] found that the MAP of pork patties at higher O_2_ concentrations resulted in a greater increase in VBN content, consistent with the results of the present study. According to the Korean Ministry of Food and Drug Safety, the permissible limit for VBN in domestic meat products is set at 20 mg% [81], but regulations for plant-based meat alternatives are currently absent. In this study, the VBN content at day 50 was 10.01 mg% for control 0 °C, 21.06 mg% for control 4 °C, 18.07 mg% for vacuum 4 °C, 22.23 mg% for MAP 0 °C, and MAP 4 °C 36.89 mg%. Considering the absence of domestic regulations on plant-based meat alternatives, caution should be exercised during consumption.

### 3.6. E-Nose

**Figure 1:**
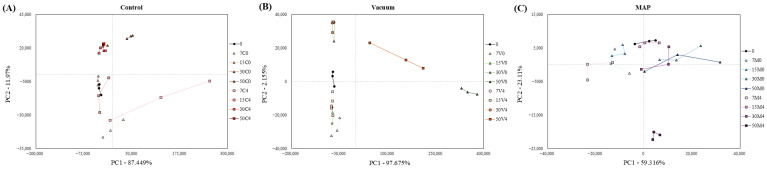

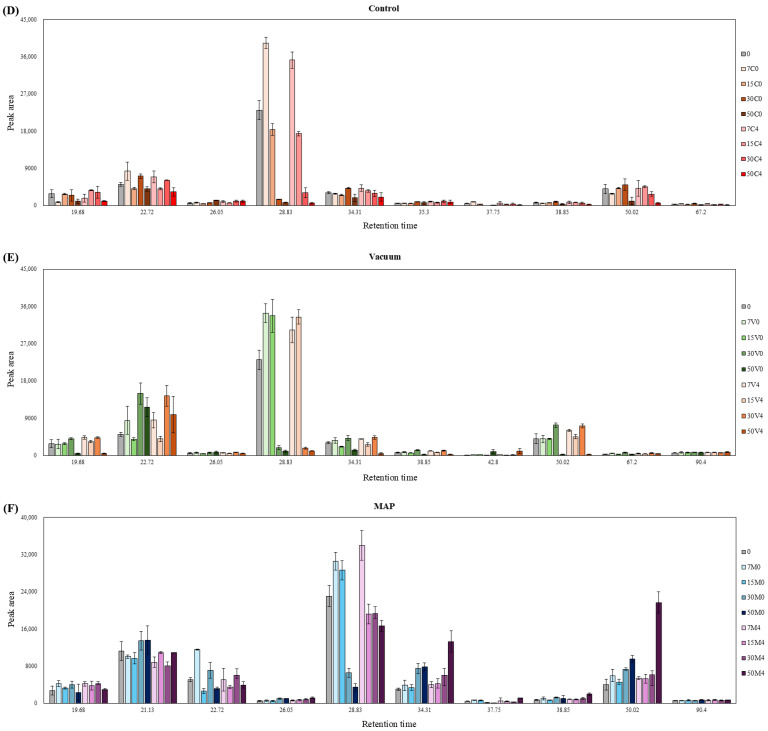
Electronic nose of sausage manufactured with shiitake mushroom mycelium. (**A**–**C**) Principal component analysis (PCA) sample distribution diagram of sausage manufactured with shiitake mushroom mycelium by electronic nose. (**D**–**F**) Predicted volatile compounds in sausage manufactured with shiitake mushroom mycelium by electronic nose. 0: day 0; 7C0: control 0 °C, day 7;15C0: control 0 °C, day 15; 30C0: control 0 °C, day 30; 50C0: control 0 °C, day 50; 7C4: control 4 °C, day 7; 15C4: control 4 °C, day 15; 30C4: control 4 °C, day 30; 50C4: control 4 °C, day 50; 7V0: vacuum 0 °C, day 7; 15V0: vacuum 0 °C, day 15; 30V0: vacuum 0 °C, day 30; 50V0: vacuum 0 °C, day 50; 7V4: vacuum 4 °C, day 7; 15V4: vacuum 4 °C, day 15; 30V4: vacuum 4 °C, day 30; 50V4: vacuum 4 °C, day 50; 7M0: MAP 0 °C, day 7; 15M0: MAP 0 °C, day 15; 30M0: MAP 0 °C, day 30; 50M0: MAP 0 °C, day 50; 7M4: MAP 4 °C, day 7; 15M4: MAP 4 °C, day 15; 30M4: MAP 4 °C, day 30; 50M4: MAP 4 °C, day 50. Retention time (RT) 19.68: acetaldehyde; RT 21.13: ethanol; RT 22.72: pentane RT 26.05: butanal; RT 28.83: 3-methylpentane; RT 34.31: (E)-but-2-enal; RT 35.3: pentan-2-one; RT 37.75: acetoin; RT 38.85: 2,3-Pentanedione; RT 42.8: (E)-2-pentenal; RT 50.02: hexanal; RT 67.2: 2,4,5-trimethylthiazole; RT 90.4: delta-nonalactone.

The e-nose results for SMMSs according to packaging method, storage temperature, and duration are shown in Figure 1. The principal component analysis results are shown in Figure 1. (A) PC1 and PC2 for the control group were 87.449% and 11.97%, respectively, whereas (B) PC1 and PC2 for the vacuum-treated group were 97.675% and 2.155%, respectively. (C) PC1 and PC2 in the MAP-treated group were 59.316% and 23.11%, respectively. Differences between the treatment groups were primarily distinguished by PC1. For the control (A), values decreased to negative values over time from day 0 to day 7 along the Y-axis and then gradually increased to positive values as the storage period progressed. (B) For the vacuum treatment group, similar to the (A) control, values decreased to negative values from day 0 to day 7 along the Y-axis and then gradually increased to positive values across all pre-treatment groups as the storage period progressed. However, at 50 days, both the vacuum 0 °C and 4 °C treatment groups showed positive values along the X-axis, indicating that a specific compound distinguished the differences between the 50-day treatment groups. (C) For the MAP-treated group, the values decreased to negative values as the storage period progressed from day 0 to 7 along the X-axis. Subsequently, all pretreated groups gradually showed positive values as the storage period continued. Furthermore, at day 50, the MAP 4 °C-treated group showed negative values along the Y-axis, unlike the other treatment groups. This difference can be distinguished based on the specific compound.

The expected compound results for SMMSs based on packaging method, storage temperature, and duration showed different patterns at 50 days for the MAP-treated group compared to the control and vacuum-treated groups, with retention time (RT) 34.31 and RT 50.02, respectively. The compound anticipated at RT 34.31, [E]-but-2-enal, is an unsaturated aldehyde found in plants and is known to be produced during the oxidation process of linolenic acid [82,83]. As the unsaturated fatty acid content decreased due to oxidation, the concentration of [E]-but-2-enal increased. These unsaturated aldehydes are toxic and adversely affect human health. Moreover, an increase in the concentration of unsaturated aldehydes, such as [E]-but-2-enal, is associated with off-flavors related to lipids and rancidity [83]. Consequently, in the MAP-treated group with high O_2_ concentration, the oxidation of unsaturated fatty acids was accelerated, resulting in a high concentration of [E]-but-2-enal on day 50. Therefore, it is judged that caution is needed when consuming it after 30 days due to toxicity and off-flavor. Hexanal, anticipated to be compound RT 50.02, belongs to the aldehyde group. It is known as a compound associated with quality deterioration during storage because it is produced by the oxidation of unsaturated fatty acids and imparts a rancid flavor [84]. Furthermore, hexanal can be oxidized to hexanoic acid or reduced to hexanol by microbial metabolism during storage, leading to a decrease in its concentration [85]. Zhu et al. [86] reported that hexanal was produced by the oxidation of unsaturated fatty acids, linoleic acid, and linolenic acid. During storage, hexanal undergoes oxidation/reduction and is converted into acids or alcohols, resulting in undetectable concentrations. In this study, concentrations decreased by day 50 in the control and vacuum-treated groups but increased in the MAP-treated group by day 50. This was attributed to the fact that, in the control and vacuum-treated groups, APC increased more rapidly than MAP, leading to more active oxidation/reduction reactions of hexanal by microorganisms during storage, thereby reducing the concentrations. Conversely, in the MAP-treated group, microbial proliferation was inhibited, reducing the hexanal oxidation/reduction reactions. Simultaneously, the continuous oxidation of unsaturated fatty acids due to the 40% O_2_ concentration led to increased hexanal formation. Therefore, the increase in hexanal over the storage period and the high hexanal concentration observed in the MAP-treated group at day 50 is judged to contribute to rancid off-flavors, which may negatively affect consumer acceptance.

### 3.7. E-Tongue

**Figure 2:**
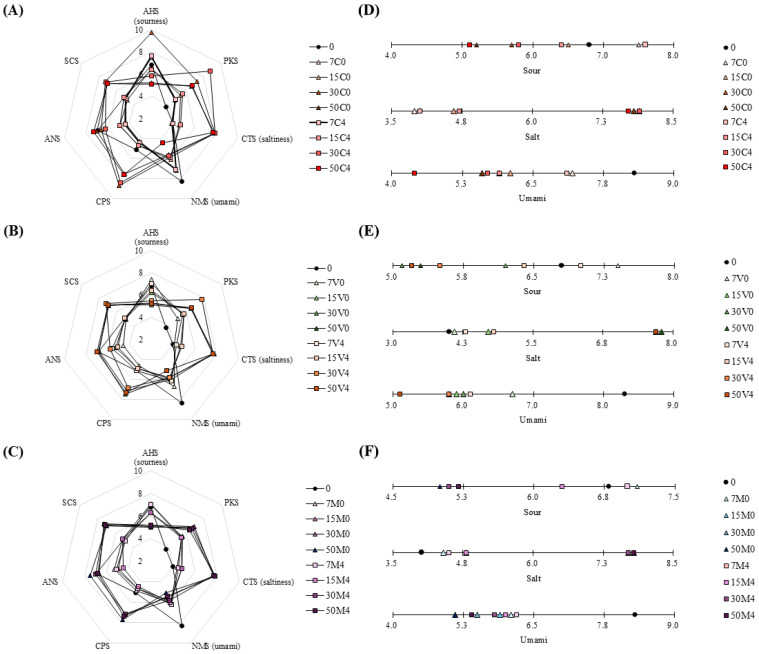
Electronic tongue of sausage manufactured with shiitake mushroom mycelium. (**A**–**C**) Changes in sensory characteristics of sausage manufactured with shiitake mushroom mycelium expressed by radar. (**D**–**F**) Changes in sensory characteristics of sausage manufactured with shiitake mushroom mycelium expressed in ranking. 0: day 0; 7C0: control 0 °C, day 7; 15C0: control 0 °C, day 15; 30C0: control 0 °C, day 30; 50C0: control 0 °C, day 50; 7C4: control 4 °C, day 7; 15C4: control 4 °C, day 15; 30C4: control 4 °C, day 30; 50C4: control 4 °C, day 50; 7V0: vacuum 0 °C, day 7; 15V0: vacuum 0 °C, day 15; 30V0: vacuum 0 °C, day 30; 50V0: vacuum 0 °C, day 50; 7V4: vacuum 4 °C, day 7; 15V4: vacuum 4 °C, day 15; 30V4: vacuum 4 °C, day 30; 50V4: vacuum 4 °C, day 50; 7M0: MAP 0 °C, day 7; 15M0: MAP 0 °C, day 15; 30M0: MAP 0 °C, day 30; 50M0: MAP 0 °C, day 50; 7M4: MAP 4 °C, day 7; 15M4: MAP 4 °C, day 15; 30M4: MAP 4 °C, day 30; 50M4: MAP 4 °C, day 50.

The e-tongue results for SMMSs according to packaging method, storage temperature, and duration are shown in Figure 2. As the storage period progressed, the sourness and umami taste decreased in the pretreated samples, whereas saltiness showed an increasing trend. Organic acids, the major metabolites in plants, impart sourness. Shiitake mushroom mycelia contain various organic acids, such as tartaric acid, oxalic acid, malic acid, and succinic acid [87,88]. However, organic acids are degraded during storage, leading to a decrease in their content [89]. Furthermore, shiitake mushroom mycelium contains flavor nucleotides, such as 5′-inosine monophosphate (5′-IMP), 5′-adenosine monophosphate, and 5′-guanosine monophosphate, which are known to impart umami flavor to meat [88,90]. Among these, 5′-IMP enhances umami flavor through interaction with glutamic acid. However, its concentration decreases due to degradation over storage time, leading to a reduction in the umami flavor [90]. Therefore, it is concluded that the decrease in organic acids and 5′-IMP content in shiitake mushroom mycelium over the storage period contributed to the reduction in sourness and umami flavor. Taste buds are sensitive to NaCl and KCl when detecting saltiness and food undergoes a relative increase in salt concentration during storage as moisture loss increases [91,92]. In this study, 1.5% NaCl was added during the production of SMMSs. As the storage period progressed, the pretreated samples exhibited an increasing trend in storage loss. Consequently, the salt concentration in shiitake mushroom mycelium-containing sausages increased, leading to increased saltiness. Saltiness is an important sensory attribute that enhances the flavor of foods; however, the World Health Organization recommends limiting salt intake to prevent diseases such as stroke and hypertension [93]. Accordingly, numerous studies have aimed to reduce NaCl content by incorporating KCl, flavor enhancers, or umami substances [94,95,96]. Therefore, the increase in saltiness over the storage period is not expected to negatively affect consumer acceptance, and any reduction in flavor due to decreased umami can be compensated by the addition of flavor enhancers.

## 4. Conclusions

This study evaluated the quality characteristics and storage stability of SMMS by varying the packaging methods, storage temperatures, and storage periods. As the storage period progressed, the moisture content of the pretreated samples decreased, whereas the protein content showed a relative increase; the ash content did not exhibit significant differences. As the storage period progressed, the pH of the pretreated samples decreased, whereas storage loss tended to increase. Furthermore, the CIE a*, Aw, APC, TBARS, and VBN values of the pretreated samples showed an increasing trend over the storage period. The e-nose results indicated that unlike the control and vacuum-packed samples, (E)-but-2-enal and hexanal levels increased in the MAP-treated samples after 50 days of storage. The electron tongue test results indicated that sourness and umami decreased, while saltiness increased in the pretreated group over the storage period. Based on the results of this study, it can be concluded that vacuum packaging under 0 °C conditions is effective for maintaining the quality and storability of SMMS. Therefore, it is expected that vacuum packaging can improve the commercial value of SMMSs by inhibiting oxidation and improving the safety and shelf life of the sausages. Meanwhile, while the MAP-treated group excelled at inhibiting aerobic microbial growth, the presence of O_2_ promoted oxidation of the sausage, adversely affecting TBARS, VBN results, and flavor characteristics. Therefore, it is judged that when packaging SMMSs in MAP, the O_2_ concentration should be reduced and replaced with an inert gas such as N_2_. This approach is expected to be applicable in practice, as it may enhance shelf life and stability by inhibiting microbial spoilage and oxidation. However, since this study did not include experiments on texture analysis, microbiological identification, or sensory evaluation, it is judged that these aspects should be addressed in future research.

## Figures and Tables

**Table 1:** Proximate composition of sausage manufactured with shiitake mushroom mycelium.

Properties (%)	Storage Day	Treatment					
		Control		Vacuum		MAP	
		0 °C	4 °C	0 °C	4 °C	0 °C	4 °C
Moisture	0	73.36 ± 0.08 ^A^	73.36 ± 0.08 ^A^	73.36 ± 0.08 ^A^	73.36 ± 0.08 ^A^	73.36 ± 0.08 ^A^	73.36 ± 0.08 ^A^
	7	72.59 ± 0.20 ^Ba^	72.51 ± 0.32 ^Ba^	72.52 ± 0.24 ^Ba^	72.52 ± 0.17 ^Ba^	72.66 ± 0.10 ^Ba^	72.42 ± 0.28 ^Ba^
	15	72.36 ± 0.16 ^Bab^	72.28 ± 0.26 ^Bb^	72.34 ± 0.41 ^Bab^	72.78 ± 0.38 ^ABa^	72.52 ± 0.19 ^Bab^	72.31 ± 0.29 ^Bab^
	30	72.57 ± 0.40 ^Ba^	72.21 ± 0.47 ^Ba^	72.47 ± 0.50 ^Ba^	72.61 ± 0.41 ^Ba^	72.42 ± 0.20 ^Ba^	72.40 ± 0.17 ^Ba^
	50	71.80 ± 0.58 ^Cab^	70.99 ± 0.42 ^Cb^	72.11 ± 0.61 ^Ba^	71.78 ± 0.64 ^Cab^	72.37 ± 0.50 ^Ba^	71.72 ± 0.36 ^Cab^
Protein	0	25.04 ± 0.09 ^B^	25.04 ± 0.09 ^B^	25.04 ± 0.09 ^C^	25.04 ± 0.09 ^C^	25.04 ± 0.09 ^B^	25.04 ± 0.09 ^B^
	7	25.76 ± 0.31 ^Aab^	25.47 ± 0.24 ^Aab^	25.32 ± 0.25 ^Bb^	25.42 ± 0.37 ^BCab^	25.85 ± 0.24 ^Aa^	25.51 ± 0.28 ^ABab^
	15	25.82 ± 0.24 ^Aa^	25.74 ± 0.26 ^Aa^	25.77 ± 0.12 ^Aa^	25.62 ± 0.19 ^ABa^	25.86 ± 0.24 ^Aa^	25.58 ± 0.66 ^Aa^
	30	25.54 ± 0.36 ^Aa^	25.77 ± 0.25 ^Aa^	25.74 ± 0.14 ^Aa^	25.91 ± 0.31 ^Aa^	25.90 ± 0.59 ^Aa^	26.03 ± 0.08 ^Aa^
	50	25.65 ± 0.25 ^Aa^	25.78 ± 0.29 ^Aa^	25.67 ± 0.09 ^Aa^	25.52 ± 0.38 ^ABa^	25.64 ± 0.19 ^Aa^	25.92 ± 0.25 ^Aa^
Ash	0	1.14 ± 0.02 ^A^	1.14 ± 0.02 ^A^	1.14 ± 0.02 ^A^	1.14 ± 0.02 ^A^	1.14 ± 0.02 ^A^	1.14 ± 0.02 ^A^
	7	1.14 ± 0.04 ^Aa^	1.14 ± 0.02 ^Aa^	1.15 ± 0.03 ^Aa^	1.14 ± 0.04 ^Aa^	1.12 ± 0.01 ^Aa^	1.13 ± 0.02 ^Aa^
	15	1.11 ± 0.01 ^Aa^	1.13 ± 0.08 ^Aa^	1.11 ± 0.08 ^Aa^	1.10 ± 0.05 ^Aa^	1.12 ± 0.06 ^Aa^	1.14 ± 0.05 ^Aa^
	30	1.13 ± 0.02 ^Aab^	1.13 ± 0.02 ^Aab^	1.14 ± 0.01 ^Aa^	1.13 ± 0.02 ^Aab^	1.13 ± 0.01 ^Aab^	1.11 ± 0.01 ^Ab^
	50	1.14 ± 0.06 ^Aa^	1.14 ± 0.03 ^Aa^	1.13 ± 0.02 ^Aa^	1.14 ± 0.02 ^Aa^	1.14 ± 0.02 ^Aa^	1.13 ± 0.03 ^Aa^

All values are mean ± SD. Means in the same column with different letters (A–C) are significantly different (*p* < 0.05). Means in the same row with different letters (a, b) are significantly different (*p* < 0.05). Control: N_2_ 78%, O_2_ 20%; MAP: Modified atmosphere packaging, CO_2_ 60%, O_2_ 40%.

**Table 2:** pH and storage loss of sausage manufactured with shiitake mushroom mycelium.

Traits	Storage Day	Treatment					
		Control		Vacuum		MAP	
		0 °C	4 °C	0 °C	4 °C	0 °C	4 °C
pH	0	6.09 ± 0.05 ^A^	6.09 ± 0.05 ^A^	6.09 ± 0.05 ^A^	6.09 ± 0.05 ^A^	6.09 ± 0.05 ^A^	6.09 ± 0.05 ^A^
	7	6.05 ± 0.01 ^Aa^	6.03 ± 0.01 ^ABa^	6.04 ± 0.01 ^Aa^	6.03 ± 0.01 ^ABa^	6.06 ± 0.05 ^Aa^	6.05 ± 0.01 ^Aa^
	15	6.03 ± 0.03 ^Aa^	6.02 ± 0.01 ^Bbc^	6.03 ± 0.01 ^Aab^	6.01 ± 0.01 ^Bbc^	6.01 ± 0.01 ^ABbc^	6.02 ± 0.00 ^Bc^
	30	6.03 ± 0.00 ^Aa^	6.03 ± 0.00 ^ABbc^	6.03 ± 0.00 ^Aa^	6.02 ± 0.00 ^ABc^	6.00 ± 0.00 ^Bd^	6.00 ± 0.00 ^Bd^
	50	5.95 ± 0.10 ^Bab^	5.87 ± 0.01 ^Cbc^	5.94 ± 0.03 ^Ba^	5.90 ± 0.04 ^Cab^	5.87 ± 0.02 ^Cb^	5.86 ± 0.01 ^Cb^
Storage loss (%)	0	0.00 ^B^	0.00 ^C^	0.00 ^C^	0.00 ^D^	0.00 ^C^	0.00 ^D^
	7	0.80 ± 0.53 ^ABd^	2.00 ± 0.40 ^Bc^	4.07 ± 0.76 ^Bb^	4.67 ± 0.50 ^Cb^	3.87 ± 0.90 ^Bb^	8.80 ± 0.53 ^Ca^
	15	1.40 ± 0.87 ^Ad^	3.13 ± 0.42 ^Ac^	6.53 ± 0.64 ^Ab^	6.73 ± 0.42 ^Bb^	4.13 ± 0.61 ^Bc^	12.33 ± 0.50 ^Ba^
	30	1.53 ± 0.42 ^Ae^	3.27 ± 0.50 ^Ad^	6.60 ± 0.92 ^Abc^	7.40 ± 0.92 ^Bb^	5.93 ± 0.70 ^Ac^	13.67 ± 0.95 ^Aa^
	50	1.67 ± 0.31 ^Af^	3.80 ± 0.20 ^Ae^	7.87 ± 0.90 ^Ac^	9.73 ± 0.61 ^Ab^	6.40 ± 0.72 ^Ad^	14.47 ± 0.76 ^Aa^

All values are mean ± SD. Means in the same column with different letters (A–D) are significantly different (*p* < 0.05). Means in the same row with different letters (a–f) are significantly different (*p* < 0.05). Control: N_2_ 78%, O_2_ 20%; MAP: Modified atmosphere packaging, CO_2_ 60%, O_2_ 40%.

**Table 3:** Color of sausage manufactured with shiitake mushroom mycelium.

Characteristics	Storage Day	Treatment					
		Control		Vacuum		MAP	
		0 °C	4 °C	0 °C	4 °C	0 °C	4 °C
CIE L*	0	65.87 ± 0.15 ^A^	65.87 ± 0.15 ^A^	65.87 ± 0.15 ^A^	65.87 ± 0.15 ^A^	65.87 ± 0.15 ^A^	65.87 ± 0.15 ^A^
	7	64.10 ± 0.35 ^Ba^	64.70 ± 0.35 ^Ba^	64.47 ± 0.93 ^Ba^	64.33 ± 0.60 ^Ba^	63.97 ± 0.15 ^Ba^	63.90 ± 0.53 ^Ba^
	15	63.40 ± 0.26 ^Cc^	63.53 ± 0.06 ^Cc^	64.57 ± 0.15 ^Ba^	64.10 ± 0.10 ^Bb^	63.87 ± 0.15 ^Bb^	63.83 ± 0.06 ^Bb^
	30	61.47 ± 0.47 ^Dc^	61.43 ± 0.12 ^Dc^	64.53 ± 0.72 ^Ba^	64.43 ± 0.55 ^Ba^	63.27 ± 0.61 ^Cb^	63.07 ± 0.76 ^BCb^
	50	61.20 ± 0.20 ^Dc^	61.13 ± 0.55 ^Dc^	64.53 ± 0.15 ^Ba^	64.07 ± 0.32 ^Ba^	63.10 ± 0.17 ^Cb^	62.67 ± 0.32 ^Cb^
CIE a*	0	3.40 ± 0.10 ^C^	3.40 ± 0.10 ^C^	3.40 ± 0.10 ^A^	3.40 ± 0.10 ^A^	3.40 ± 0.10 ^D^	3.40 ± 0.10 ^C^
	7	3.40 ± 0.17 ^Ca^	3.63 ± 0.23 ^Ba^	3.40 ± 0.17 ^Aa^	3.43 ± 0.06 ^Aa^	3.60 ± 0.10 ^CDa^	3.53 ± 0.15 ^Ca^
	15	3.50 ± 0.10 ^BCb^	3.73 ± 0.06 ^Ba^	3.33 ± 0.06 ^Ab^	3.43 ± 0.21 ^Ab^	3.80 ± 0.10 ^BCa^	3.93 ± 0.06 ^Ba^
	30	3.70 ± 0.10 ^Bab^	3.87 ± 0.29 ^Ba^	3.30 ± 0.10 ^Ac^	3.47 ± 0.12 ^Abc^	3.93 ± 0.12 ^Ba^	4.03 ± 0.32 ^Ba^
	50	4.40 ± 0.10 ^Ab^	5.17 ± 0.15 ^Aa^	3.50 ± 0.10 ^Ac^	3.53 ± 0.06 ^Ac^	4.43 ± 0.25 ^Ab^	4.93 ± 0.15 ^Aa^
CIE b*	0	16.57 ± 0.29 ^A^	16.57 ± 0.29 ^A^	16.57 ± 0.29 ^A^	16.57 ± 0.29 ^A^	16.57 ± 0.29 ^A^	16.57 ± 0.29 ^A^
	7	14.57 ± 0.92 ^Bb^	14.63 ± 0.45 ^Bb^	14.43 ± 0.40 ^Bb^	14.47 ± 0.25 ^Bb^	16.20 ± 0.10 ^Aa^	16.23 ± 0.15 ^Aa^
	15	14.60 ± 0.17 ^Bb^	14.53 ± 0.32 ^Bb^	14.67 ± 0.15 ^Bb^	14.60 ± 0.46 ^Bb^	16.43 ± 0.74 ^Aa^	16.33 ± 0.29 ^Aa^
	30	14.83 ± 0.38 ^Bb^	14.80 ± 0.36 ^Bb^	14.73 ± 0.15 ^Bb^	14.77 ± 0.83 ^Bb^	16.33 ± 0.70 ^Aa^	16.13 ± 0.76 ^Aa^
	50	14.77 ± 0.51 ^Bb^	14.87 ± 0.32 ^Bb^	14.83 ± 0.31 ^Bb^	14.87 ± 0.25 ^Bb^	16.80 ± 0.10 ^Aa^	16.80 ± 0.10 ^Aa^

All values are mean ± SD. Means in the same column with different letters (A–D) are significantly different (*p* < 0.05). Means in the same row with different letters (a–c) are significantly different (*p* < 0.05). Control: N_2_ 78%, O_2_ 20%; MAP: Modified atmosphere packaging, CO_2_ 60%, O_2_ 40%.

**Table 4:** Aw, APC (log CFU/g), TBARS (mg MDA/kg), and VBN (mg%) of sausage manufactured with shiitake mushroom mycelium.

Traits	Storage Day	Treatment					
		Control		Vacuum		MAP	
		0 °C	4 °C	0 °C	4 °C	0 °C	4 °C
Aw	0	0.81 ± 0.00 ^C^	0.81 ± 0.00 ^C^	0.81 ± 0.00 ^C^	0.81 ± 0.00 ^D^	0.81 ± 0.00 ^D^	0.81 ± 0.00 ^D^
	7	0.84 ± 0.00 ^Bab^	0.84 ± 0.00 ^Ba^	0.83 ± 0.00 ^Bc^	0.84 ± 0.00 ^Ca^	0.83 ± 0.00 ^Cbc^	0.84 ± 0.00 ^Ca^
	15	0.84 ± 0.00 ^Aa^	0.84 ± 0.00 ^Aa^	0.84 ± 0.00 ^Bc^	0.84 ± 0.00 ^Bb^	0.84 ± 0.00 ^Bb^	0.84 ± 0.00 ^Bb^
	30	0.84 ± 0.00 ^Aa^	0.84 ± 0.00 ^Aa^	0.84 ± 0.00 ^Ad^	0.84 ± 0.00 ^ABc^	0.84 ± 0.00 ^Abc^	0.84 ± 0.00 ^Aab^
	50	0.84 ± 0.00 ^Aa^	0.84 ± 0.00 ^Aab^	0.84 ± 0.00 ^Ac^	0.84 ± 0.00 ^Aabc^	0.84 ± 0.00 ^Abc^	0.84 ± 0.00 ^Aabc^
APC	0	0.18 ± 0.52 ^B^	0.18 ± 0.52 ^B^	0.18 ± 0.52 ^B^	0.18 ± 0.52 ^B^	0.18 ± 0.52 ^B^	0.18 ± 0.52 ^B^
	7	0.37 ± 0.68 ^Ba^	0.55 ± 0.76 ^Ba^	0.18 ± 0.52 ^Ba^	0.37 ± 0.68 ^Ba^	0.18 ± 0.52 ^Ba^	0.18 ± 0.52 ^Ba^
	15	0.51 ± 0.72 ^Bab^	1.00 ± 0.85 ^Ba^	0.33 ± 0.62 ^Bab^	0.63 ± 0.70 ^Bab^	0.16 ± 0.46 ^Bb^	0.51 ± 0.65 ^Bab^
	30	2.10 ± 0.17 ^Ab^	3.68 ± 0.52 ^Aa^	1.50 ± 1.00 ^Ab^	2.00 ± 0.00 ^Ab^	0.25 ± 0.71 ^Bc^	0.54 ± 1.00 ^Bc^
	50	2.59 ± 0.36 ^Ab^	3.90 ± 0.82 ^Aa^	2.50 ± 0.71 ^Ab^	2.65 ± 0.49 ^Ab^	2.00 ± 0.00 ^Ab^	2.00 ± 0.00 ^Ab^
TBARS	0	0.41 ± 0.01 ^C^	0.41 ± 0.01 ^C^	0.41 ± 0.01 ^B^	0.41 ± 0.01 ^C^	0.41 ± 0.01 ^C^	0.41 ± 0.01 ^D^
	7	0.46 ± 0.01 ^Bb^	0.46 ± 0.01 ^Bb^	0.46 ± 0.01 ^Ab^	0.46 ± 0.01 ^Bb^	0.53 ± 0.01 ^Ba^	0.54 ± 0.01 ^Ca^
	15	0.47 ± 0.01 ^Bb^	0.49 ± 0.03 ^Bb^	0.46 ± 0.02 ^Ab^	0.48 ± 0.01 ^Bb^	0.56 ± 0.03 ^Ba^	0.59 ± 0.02 ^BCa^
	30	0.50 ± 0.04 ^ABbc^	0.54 ± 0.02 ^Ab^	0.46 ± 0.01 ^Ac^	0.47 ± 0.02 ^Bc^	0.60 ± 0.02 ^Aa^	0.64 ± 0.03 ^Ba^
	50	0.53 ± 0.03 ^Ac^	0.56 ± 0.01 ^Abc^	0.50 ± 0.04 ^Ac^	0.52 ± 0.02 ^Ac^	0.63 ± 0.01 ^Ab^	0.75 ± 0.10 ^Aa^
VBN	0	0.37 ± 0.17 ^D^	0.37 ± 0.17 ^D^	0.37 ± 0.17 ^C^	0.37 ± 0.17 ^D^	0.37 ± 0.17 ^D^	0.37 ± 0.17 ^D^
	7	2.17 ± 0.13 ^Ca^	2.24 ± 0.22 ^Ca^	2.09 ± 0.13 ^Ba^	2.09 ± 0.13 ^Ca^	2.17 ± 0.13 ^Ca^	2.31 ± 0.13 ^Ca^
	15	2.24 ± 0.22 ^Cc^	2.61 ± 0.13 ^Ca^	2.09 ± 0.13 ^Bc^	2.31 ± 0.13 ^Cbc^	2.54 ± 0.13 ^Cab^	2.65 ± 0.06 ^Ca^
	30	3.88 ± 0.13 ^Bd^	7.47 ± 0.34 ^Bc^	2.39 ± 0.13 ^Be^	3.73 ± 0.34 ^Bd^	8.29 ± 0.22 ^Bb^	30.02 ± 0.45 ^Ba^
	50	10.01 ± 0.47 ^Ae^	21.06 ± 0.22 ^Ac^	4.26 ± 0.22 ^Af^	18.07 ± 0.47 ^Ad^	22.33 ± 0.91 ^Ab^	36.89 ± 0.47 ^Aa^

All values are mean ± SD. Means in the same column with different letters (A–D) are significantly different (*p* < 0.05). Means in the same row with different letters (a–f) are significantly different (*p* < 0.05). Control: N_2_ 78%, O_2_ 20%; MAP: Modified atmosphere packaging, CO_2_ 60%, O_2_ 40%.

## Data Availability

The original contributions presented in this study are included in the article. Further inquiries can be directed to the corresponding author.

## Abbreviations

SMMS	Shiitake mushroom mycelium sausage
MAP	Modified atmosphere packaging
RT	Retention time
Aw	Water activity
APC	Aerobic plate count
TBARS	Thiobarbituric acid reactive substances
VBN	Volatile basic nitrogen
e-nose	Electronic nose
e-tongue	Electronic tongue
0	Day 0
7C0	Control 0 °C, day 7
15C0	Control 0 °C, day 15
30C0	Control 0 °C, day 30
50C0	Control 0 °C, day 50
7C4	Control 4 °C, day 7
15C4	Control 4 °C, day 15
30C4	Control 4 °C, day 30
50C4	Control 4 °C, day 50
7V0	Vacuum 0 °C, day 7
15V0	Vacuum 0 °C, day 15
30V0	Vacuum 0 °C, day 30
50V0	Vacuum 0 °C, day 50
7V4	Vacuum 4 °C, day 7
15V4	Vacuum 4 °C, day 15
30V4	Vacuum 4 °C, day 30
50V4	Vacuum 4 °C, day 50
7M0	MAP 0 °C, day 7
15M0	MAP 0 °C, day 15
30M0	MAP 0 °C, day 30
50M0	MAP 0 °C, day 50
7M4	MAP 4 °C, day 7
15M4	MAP 4 °C, day 15
30M4	MAP 4 °C, day 30
50M4	MAP 4 °C, day 50
